# Personal Devices to Monitor Physical Activity and Nutritional Intake After Colorectal Cancer Surgery: Feasibility Study

**DOI:** 10.2196/40352

**Published:** 2022-12-13

**Authors:** Manouk J W van der Linden, Lenny M W Nahar van Venrooij, Emiel G G Verdaasdonk

**Affiliations:** 1 Department of Dietetics Jeroen Bosch Hospital ’s-Hertogenbosch Netherlands; 2 Jeroen Bosch Academy Research Jeroen Bosch Hospital ’s-Hertogenbosch Netherlands; 3 Department of Surgery Jeroen Bosch Hospital ’s-Hertogenbosch Netherlands

**Keywords:** eHealth, fitness trackers, diet records, colorectal neoplasm, colorectal cancer, surgery, self management, patient care, physical activity, tracking, activity tracking, self-monitoring, feasibility, usability

## Abstract

**Background:**

The use of self-monitoring devices is promising for improving perioperative physical activity and nutritional intake.

**Objective:**

This study aimed to assess the feasibility, usability, and acceptability of a physical activity tracker and digital food record in persons scheduled for colorectal cancer (CRC) surgery.

**Methods:**

This observational cohort study was conducted at a large training hospital between November 2019 and November 2020. The study population consisted of persons with CRC between 18- and 75 years of age who were able to use a smartphone or tablet and scheduled for elective surgery with curative intent. Excluded were persons not proficient in Dutch or following a protein-restricted diet. Participants used an activity tracker (Fitbit Charge 3) from 4 weeks before until 6 weeks after surgery. In the week before surgery (preoperative) and the fifth week after surgery (postoperative), participants also used a food record for 1 week. They shared their experience regarding usability (system usability scale, range 0-100) and acceptability (net promoter score, range –100 to +100).

**Results:**

In total, 28 persons were included (n=16, 57% male, mean age 61, SD 8 years), and 27 shared their experiences. Scores regarding the activity tracker were as follows: preoperative median system usability score, 85 (IQR 73-90); net promoter score, +65; postoperative median system usability score, 78 (IQR 68-85); net promotor score, +67. The net promoter scores regarding the food record were +37 (preoperative) and–7 (postoperative).

**Conclusions:**

The perioperative use of a physical activity tracker is considered feasible, usable, and acceptable by persons with CRC in this study. Preoperatively, the use of a digital food record was acceptable, and postoperatively, the acceptability decreased.

## Introduction

There is a growing body of literature that recognizes the importance of physical fitness in colorectal cancer (CRC) surgery. Higher levels of physical activity have been associated with improved outcomes such as decreased cancer mortality and recurrence rates [1,2]. After surgery, activity levels are low, and a decline in physical function and the incidence of psychological distress can negatively impact recovery [3,4]. A higher level of preoperative physical activity is associated with health-related quality of life and reduced adverse perioperative outcomes, such as complications, length of hospital stay, and readmissions [5-8].

Given the potential to optimize physical fitness during the waiting period before surgery, the focus has shifted from rehabilitation to prehabilitation. Prehabilitation comprises the process of enhancing the functional and mental capacity of persons to buffer against potential adverse effects of a major stressor, such as surgery [9]. Prehabilitation programs incorporate exercise training with enhanced medical and psychological status [10]. Another important component of prehabilitation is the optimization of nutritional status with a focus on adequate protein intake [11]. Several studies have shown that multimodal prehabilitation can improve the physical fitness of persons with surgical CRC, although the effect on clinical outcomes remains less clear [12-15]. Persons feel the need to physically prepare for surgery and enjoy the experience of prehabilitation [16]. However, such programs could be time-consuming and labor-intensive, and most have suboptimal participant adherence rates [16,17].

In the last decade, interest in the use of physical activity trackers (PATs) in health care has increased. PATs provide automatic dynamic data tracking and can be linked to smartphones and other relevant fitness applications such as digital food records (DFRs). This ensures immediate availability of point-of-care data, such as steps per day and protein intake, with the ability to generate automated goal-directed alerts to users. PATs are used in persons with chronic diseases to improve physical fitness and are increasingly popular in oncology practice [18].

Several studies conclude that the use of PATs is feasible to objectively assess physical activity in CRC surgery [19-29]. Preliminary evidence suggests that physical activity measured by PATs is associated with postoperative outcomes after surgery [30-32]. However, questions have been raised about the suitability of PATs for this population and not much is known about persons’ experiences. Furthermore, the combined monitoring of physical activity and protein intake with digital appliances is understudied.

Through this feasibility study, we aimed to assess the usability and persons' satisfaction regarding a robust commercially available PAT (Fitbit Charge 3; Fitbit, Inc.) in CRC surgery. We also examined the person’s experience with a digital food record (DFR) to monitor nutritional intake. Additionally, we sought to obtain data on physical activity and protein intake in the perioperative period. Clinical outcomes were compared based on whether or not physical activity and protein intake goals were achieved.

## Methods

### Study Design and Setting

This observational cohort study was conducted at a large nonacademic training hospital (the Jeroen Bosch hospital, 's-Hertogenbosch) in The Netherlands from November 2019 to November 2020. In 2018, more than 250 new persons with CRC were treated at this hospital, nearly 180 of whom were eligible for curative surgery. In this hospital, the perioperative care in CRC surgery is embedded in the enhanced recovery after surgery pathways. All persons with CRC received written information about physical activity and nutrition during the perioperative period from the clinical nurse specialist. To support the usual care, participants in this study were provided a Fitbit Charge 3 to wear up to 4 weeks prior to surgery until 6 weeks after surgery on the wrist of the nondominant hand. The Fitbit was paired with the person’s smartphone or tablet, or with a borrowed tablet from the hospital if the person does not have the compatible equipment, by the Fitbit app. This app automatically provides daily statistics on physical activity and sends a weekly report to the participants by email. Participants were asked to share these weekly reports with the researcher. To monitor their nutritional intake, participants filled in a DFR, 1 week prior to surgery and the last week of the study 5 weeks after surgery.

### Participants

The study population consisted of persons with colorectal carcinoma scheduled to undergo elective surgery with curative intent. Persons between 18 and 75 years of age were included if they were able to use a smartphone or tablet that is compatible with the tracker. Persons were excluded if they were not proficient in Dutch or following a protein-restricted diet on the advice of a medical specialist.

### Recruitment

**Figure 1:**
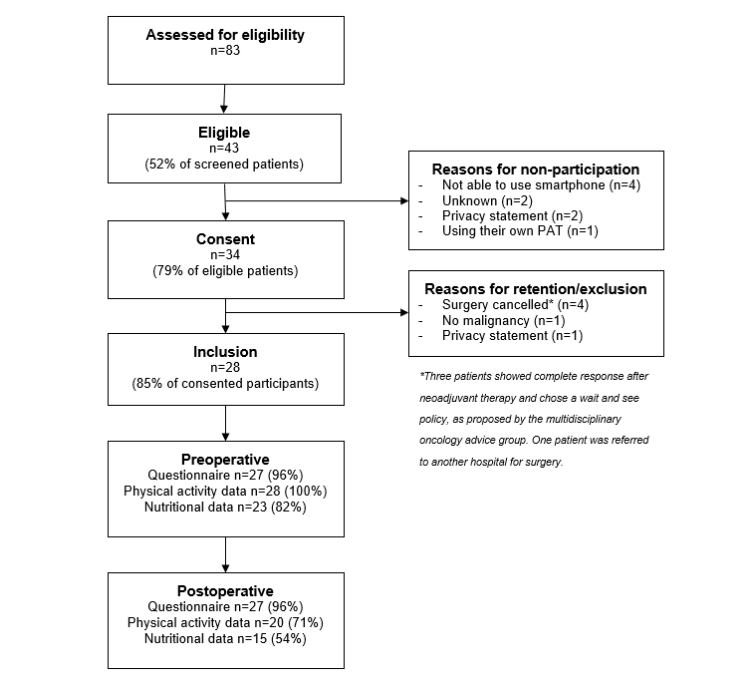
The flow of patients with colorectal cancer participating in the study. PAT: physical activity tracker.

Convenience sampling was used in which the clinical nurse specialist screened the eligibility of persons with CRC scheduled for elective and curative surgery during her consultation. Those eligible were given a study information sheet and asked permission to share contact details with the researcher. The researcher contacted people by telephone to discuss the content of the study. If a person was interested, eligibility was confirmed by the researcher and an information and consent form, approved by the local medical ethics committee, was sent. An appointment was planned with the researcher at a mutually convenient time, preferably in combination with other hospital appointments for the person. Written consent was obtained and the PAT was programmed and demonstrated in person by the researcher. Figure 1 shows the flow of participants.

### Variables

#### Feasibility, Usability, and Acceptability

Rates on screening, eligibility, consent, inclusion, and completion were collected. In an online questionnaire, persons were asked to share their views on the usability and acceptability of the Fitbit and the DFR. The usability of the Fitbit was assessed by the system usability scale (SUS), consisting of 10 statements regarding the usability of an electronic device or system that participants can rate on a 5-point Likert scale. The Fitbit was considered usable if the mean SUS were higher than 68 [33]. Acceptability regarding the Fitbit and the DFR was measured using the customer satisfaction score (CSAT) and the net promotor score (NPS). The CSAT is a score where respondents indicate acceptability using a 5-point Likert scale answering the question: “How satisfied were you with your experience?” This score focuses mainly on short-term acceptability, whereas the NPS focuses more on the long term. The NPS is calculated based on responses to the question: “How likely is it that you would recommend our company/product/ service to a friend or colleague?” using a scale of 0-10. The percentage of detractors (answering with 1-6) was subtracted from the percentage of promoters (answering with 9 or 10). Passive scores (answering with 7 or 8) were not counted. An NPS could be as low as –100 or as high as +100. A positive total NPS was considered acceptable [34].

#### Physical Activity

Physical activity was monitored using the Fitbit Charge 3, which has a visual display on the bracelet for monitoring activity progress. The weekly number of steps provided by the reports of the Fitbit app was monitored up to 4 weeks prior to surgery until 6 weeks after surgery. Other information regarding physical activity such as the number of floors climbed, calories burned, and active minutes was visible to participants but outside the scope of this study. This wrist-worn commercially available activity tracker is an objective person-generated measure of physical activity, as it has generally high validity and reliability for measuring daily step count [35-37]. At inclusion, persons were instructed to achieve at least 7500 steps per day, which seems to be a relevant goal as reflected upon research in this population [38,39]. No additional advice or incentives were given by researchers or caregivers during the study concerning physical activity other than within usual care. There were no implications on whether or not daily step goals were achieved.

#### Protein Intake

Protein intake in grams per day was measured using a DFR of The Netherlands Nutrition Centre called “Mijn Eetmeter.” In this tool, users select and log foods from the Netherlands Food Information Resource (NethFIR) database, maintained and updated regularly by the Dutch National Institute for Public Health and the Environment (RIVM), containing macronutrient and micronutrient data of over 90,000 food items. This DFR is freely available to the public and is featured with a barcode scanner, options to add new foods, and remember favorite foods, and the ability to export nutritional data. Participants filled in their daily consumed foods and monitored their protein intake twice for 7 consecutive days. This method has similar validity and reliability when compared to conventional methods to assess dietary protein intake [40-42]. The DFR provided a weekly overview of consumed foods including daily protein intake. Other data on macronutrient and micronutrient levels were visible for participants but outside the scope of this study.

Participants were instructed to consume 1.2-1.5 g of protein per kilogram of body weight per day, with correction for underweight (BMI<20 kg/m^2^) and overweight (BMI>27.5 kg/m^2^), if necessary. This is considered the optimal amount for persons with CRC in the perioperative period [43,44]. All persons received an example daily menu from the clinical nurse specialist, which approximates individual protein requirements. Participants were advised to consume foods available in food stores that are, per definition, considered safe; no protein supplements were advised. No additional advice or incentives were given by researchers or caregivers during the study concerning protein intake other than within usual care. There were no implications on whether or not protein intake was adequate. Protein intake was considered adequate when participants ingest at least 1.2 g per kilogram body weight.

#### Person Characteristics and Clinical Outcomes

Person characteristics such as age (in years), sex (male/female), BMI (in kg/m^2^), tumor location (rectum/colon), tumor stadium (I/II/III/IV), neoadjuvant therapy (non/chemotherapy/ chemo-radiation/other), and surgical technique (laparoscopic/open) were collected. All participants are Dutch residents; further information regarding ethnicity is unknown. Clinical outcomes, measured 90 days after discharge, included the length of hospital stay (in days) in comparison with the expected number of hospital days in the care paths for colon (4 days) or rectal (6 days) cancer surgery. Data have been dichotomized (expected or longer than expected hospital stay compared with the care paths). The occurrence of complications (yes or no) and unplanned readmissions (yes or no) after hospital discharge was measured. Finally, “Textbook Outcome” was used as a composite measure of clinical process indicators [45]. Textbook outcome is realized for persons for whom all desired short-term health indicators (expected hospital stay, no complications, and no unplanned readmissions) are met.

#### Study Size

The aim of the study was not to provide an estimate of the treatment effect, so there was no formal sample size calculation. The estimation was made to recruit 30 persons over 6 months, based on the number of persons with CRC eligible for curative surgery in the previous year, clinical estimates of the number of persons eligible for inclusion (50%), and the estimated recruitment rate (80%). Due to the COVID-19 pandemic, nonmedically necessary care was delayed, and the inclusion period was extended to a total of 12 months.

### Statistical Methods

Descriptive statistics have been used to summarize all variables using SPSS Statistics v25 (IBM Corp). To test for differences in baseline characteristics and clinical outcomes between persons who achieved their goals on protein intake and physical activity and persons who did not achieve their goals, the Fisher exact test for categorical data and the Mann-Whitney *U* test for not normally distributed continuous data were used. A 2-sided *P* value of <.05 was considered statistically significant. Reporting is consistent with the STROBE (Strengthening the Reporting of Observational Studies in Epidemiology) statement for observational research [46].

#### Ethics Approval

This study was submitted for approval to the Medical Research Committee Brabant, who confirmed that the Medical Research Involving Human Subjects Act did not apply. The data were prospectively collected by applying the most recent version (version 7, October 2013) of the Declaration of Helsinki and the Guidelines for Good Clinical Practice.

## Results

### Participants

From November 2019 to November 2020, 144 persons with CRC underwent elective curative surgery. Inclusion for participation in this study was halted between March 16 and July 1 due to the measures surrounding COVID-19.

Figure 1 shows the flow of participants. A total of 28 persons were included. Although not statistically significant, the persons who declined tended to be older (*P*=.05) compared to included persons. There was no difference in gender (*P*=.45).

Table 1 displays the baseline characteristics, divided into whether or not participants reached their goals. In 5 (18%) cases, this division could not be made because of missing dietary food record data. Persons who reached their goals achieved a mean daily number of steps ≥7500 and ≥1.2 gram of protein per kilogram (adjusted) body weight per day. The participants who reached both goals preoperatively were more often female (n=9, 69% vs n=2, 20%; *P*=.04) and had lower BMI (24 kg/m^2^, IQR 22-27 vs 27 kg/m^2^, IQR 24-28; *P*=.03) compared to participants who did not reach their goals.

**Table 1:** Baseline characteristics and clinical outcomes.

Characteristics			Total (N=28)		Goals^a^ achieved (n=13)		Goals not achieved (n=10)		*P* value	
Age (years), median (IQR; min-max)		61 (15; 47-73)		57 (15; 49-73)		59 (14; 49-73)		.95		
Sex (male), n (%)		16 (57)		4 (31)		8 (80)		.04^b^		
BMI, kg/m^2^, median (IQR; min-max)		26 (4; 19-37)		24 (5; 19-30)		27 (4; 24-37)		.03^b^		
**ASA^c^ index, n (%)**										.49
	I	4 (14)		2 (15)		0 (0)				
	II	23 (82)		10 (77)		10 (100)				
	III	1 (4)		1 (8)		0 (0)				
**Tumor location**										.67
	Colon	19 (68)		8 (62)		8 (80)				
	Rectum	7 (25)		3 (23)		2 (20)				
	Both	2 (7)		2 (15)		0 (0)				
**TNM^d^ classification**										.63
	I	4 (14)		2 (15)		2 (20)				
	II	8 (29)		5 (39)		2 (20)				
	III	14 (50)		6 (46)		5 (50)				
	IV	2 (7)		0 (0)		1 (10)				
**Neoadjuvant treatment**										.67
	None	21 (75)		11 (85)		6 (60)				
	Chemotherapy	2 (7)		1 (8)		1 (10)				
	Radiotherapy	1 (4)		0 (0)		1 (10)				
	Chemo-radiation	4 (14)		1 (8)		2 (20)				
**Surgical technique**										.44
	Laparoscopic	27 (97)		13 (100)		9 (90)				
	Open	1 (4)		0 (0)		1 (10)				
LOS^e^ longer than expected			12 (43)		4 (31)		5 (50)		.42	
Complications			6 (21)		1 (8)		4 (40)		.13	
Unplanned readmissions			2 (7)		0 (0)		1 (10)		.44	
Textbook outcome			16 (57)		9 (69)		5 (50)		.42	

^a^Goals based on 1.2 grams of protein/kilogram of body weight/day and ≥7500 steps per day.

^b^A 2-sided *P* value of <.05 was considered statistically significant.

^c^ASA: American Society of Anesthesiologists.

^d^TNM: Tumor, Node, Metastasis classification

^e^LOS: length of hospital stay.

### Usability and Acceptability

The usability of the Fitbit was assessed in the week prior to surgery and 5 weeks after surgery, with a median SUS of 85 (IQR 73-90) and 78 (IQR 68-85), respectively. Acceptability of the Fitbit was scored on the NPS with preoperative having 69% (n=18 answering with 9 or 10) promotors and 4% (n=1 answering with 6) detractors. Postoperatively, the percentage of promotors was 74% (n=20 answering with 9 or 10) and 7% detractors(n=2 answering with 3 or 4). Therewith, the NPS was preoperative +65 and postoperative +67. Acceptability of the DFR was scored on the NPS with a preoperative score of +37 based on 52% (n=14 answering with 9 or 10) being promotors and 15% (n=4 answering with 5 or 6) being detractors. Postoperatively, the NPS score was –7 based on 30% (n=8 answering with 9 or 10) promotors and 37% (n=10 answering with 2 to 6) detractors. Other scores regarding the acceptability of the Fitbit and DFR are presented in Table 2. There was room for free comments in the web-based questionnaire, and 22 (79%) participants used this option. In 14 cases, the feedback focused on the DRF, with most participants (n=12) identifying drawbacks or areas for improvement. In particular, the complexity of the app used was frequently mentioned.

**Table 2:** Acceptability scores for the use of a physical activity tracker and digital food record.

		Physical activity tracker		Digital food record	
		Preoperative	Postoperative	Preoperative	Postoperative
**Scores (range 1-5),** **median (IQR; min-max)**					
	Customer satisfaction score	5 (1; 3-5)	4 (1; 2-5)	4 (0; 3-5)	3 (2; 2-5)
	The tool provides insight	5 (1; 3-5)	5 (1; 2-5)	5 (1; 2-5)	4 (1; 2-5)
	The tool stimulates to reach goal	4 (2; 1-5)	4 (2; 1-5)	4 (2; 3-5)	4 (2; 2-5)

### Physical Activity

**Figure 2:**
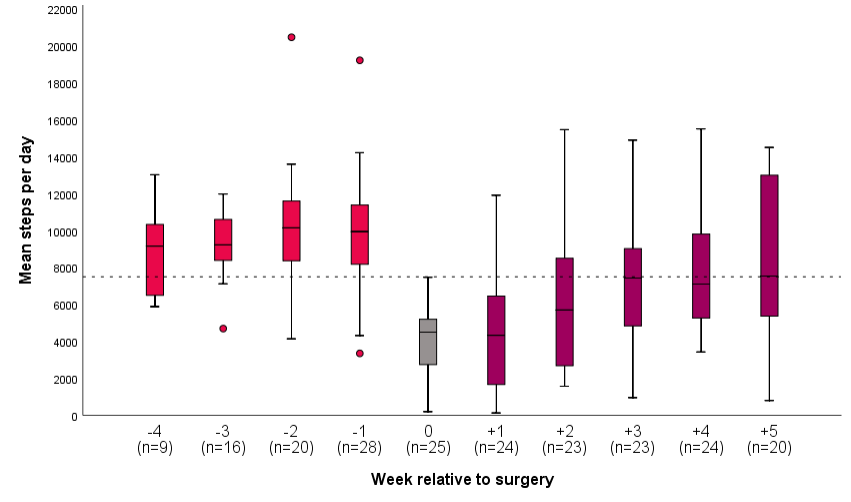
Perioperative course of physical activity in patients with colorectal cancer using a physical activity tracker. The dotted line represents the daily goal of 7500 steps per day.

Due to surgery planning, not all participants started measuring their physical activity 4 weeks before the surgery. In the week before surgery (preoperative), all participants had complete data on physical activity, and 79% (n=22) reached a mean number of steps ≥7500. A total of 14 (50%) participants provided data on physical activity in all postoperative weeks. Figure 2 shows the perioperative course of physical activity, expressed in the median number of steps per day.

### Protein Intake

**Figure 3:**
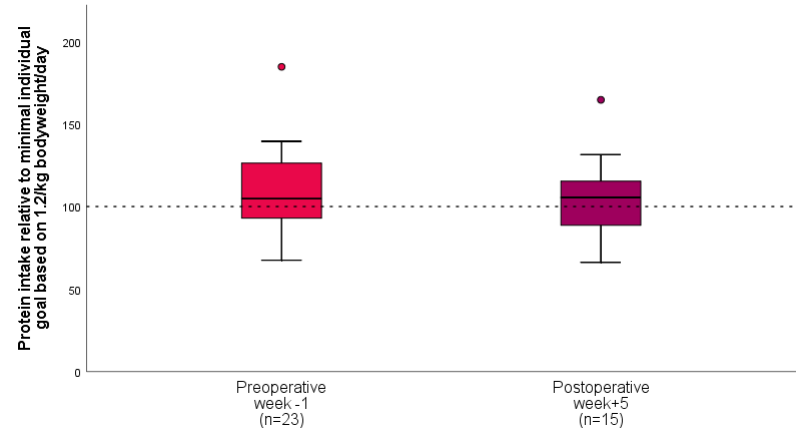
Perioperative course of protein intake in patients with colorectal cancer using a digital food record. The dotted line represents the daily protein goal.

Preoperatively, 23 (82%) participants provided data on their mean protein intake during the week before surgery, and 16 (70%) participants reached ≥100% of their individual protein intake goal. Postoperatively, 15 (54%) participants provided data on their mean protein intake during the fifth week after surgery, and 10 (67%) participants reached their goal. Figure 3 shows the median protein intake of participants relative to their minimal individual goal based on 1.2 g per kilogram body weight per day.

### Clinical Outcome

Table 1 summarizes clinical outcomes, divided into whether or not participants reached their goals. Persons who achieved ≥7500 steps per day and ≥1.2 g protein per kilogram body weight per day appeared to have lower rates of prolonged hospital stay (n=4, 31% vs n=5, 50%), complications (n=1, 8% vs n=4, 40%) and unplanned readmissions (n=0, 0% vs n=1, 10%). However, the differences were not statistically significant. The product of all clinical indicators resulted in the number and proportion of persons for whom all desired outcomes were realized and thereby a “Textbook Outcome” was achieved. The proportion of persons with a “Textbook Outcome” tended to be higher for persons who achieved their goals (n=9, 69% vs n=5, 50%; *P*=.42).

## Discussion

### Principal Findings

This study describes the feasibility, usability, and acceptability of a commercially available PAT and DFR in CRC surgery. Persons reported high scores for usability and acceptability of a PAT using the SUS, NPS, and CSAT scales. We found high compliance and high adherence rates to daily step goals in the perioperative period. The acceptability regarding the DFR was lower compared to the PAT; it was found acceptable in the preoperative setting only. The compliance rate for using the DFR was acceptable in the preoperative period with adequate protein intake in most persons, but the compliance rate dropped after surgery. Although not statistically significant, clinical outcomes appeared to benefit persons who achieved ≥7500 steps per day and ≥1.2 g protein per kilogram body weight per day.

### Comparison to Prior Work

Few studies have examined persons’ experiences regarding PATs in people who have received surgery. In a perioperative eHealth program with multiple components, den Bakker et al [21] used qualitative person feedback as well as a scale ranging from 0 to 10 to assess persons' with CRC attitudes. Participants were positive about the use of a PAT and stated that it was a good way to reflect on their level of activity, and the use motivated them to be physically active. Grimes et al [22] found high acceptability of wearing a wrist-worn accelerometer in 35 perioperative older adults, measured using a visual analogue scale questionnaire. Jonker et al [24] determined the feasibility, usability, and acceptability of the perioperative use of their mobile app and activity tracker in older persons with surgical cancer. Their scores on usability (SUS) and acceptability (NPS) were lower compared to the scores in our population, which might be related to a higher mean age of participants in their study.

The preoperative median step count in our study is comparable with other studies using PATs in persons with CRC [19,23,28]. In free-living, healthy, older adults, the reported daily number of steps ranges from around 2000 to 9000 [47]. For persons with cancer, and persons with other chronic conditions or those living with a disability, the expected range lowers to 1200-8800 steps per day [48]. Most persons in our study met the daily step goal comparable with recommended physical activity levels for persons with cancer [1] and the general population [49] prior to surgery. As expected, postoperatively, the median number of steps dropped to approximately 4300 steps/day 1 week after surgery, which is comparable to habitual steps/day in persons with heart and vascular diseases [48]. In the phase of recovery, physical activity increased, and 35% of the persons returned to the preoperative daily number of steps. In our study, the majority of persons underwent laparoscopic surgery. Functional recovery may differ between traditional open surgery and minimally invasive surgery. Due to the small number of participants (n=1) who underwent an open procedure (n=1), this could not be sufficiently explored in this study. A randomized blinded study on this topic found no difference in functional recovery after open versus laparoscopic colonic resection [50]. In line with our results, Nakajima et al [31] noted that persons with CRC with a low activity level were significantly older and had a higher rate of major complications.

Participants in our study considered the use of a DFR acceptable in the preoperative setting. This is comparable with other nutritional apps to promote a healthy lifestyle, with better scores for apps with options to memorize recent and favorite foods and a range of household and metric measures that increased the ease of self-monitoring of food intake [51]. In our study, the compliance rate was 82% (n=23) in the week prior to surgery with 70% (n=16) of participants reaching the recommended daily protein intake. After surgery, only 54% (n=15) completed their food record, of whom 67% (n=10) reached their goal. In addition to the compliance rate, the acceptability scores (CSAT and NPS) were also higher in the preoperative phase compared to the postoperative scores. This endorses the hypothesis of Grimes et al [22], who suggest that the preoperative setting may be a unique period in which behavioral interventions are more likely to be successful, possibly due to the well-defined end point (surgical procedure) and the motivation that good nutritional status can affect the surgical outcome. Although the compliance for both tools is higher in the preoperative period compared to the postoperative period, it is striking that the difference is more pronounced for the DFR. This may partly be explained by the fact that data on nutritional intake must be entered by the participants in the DFR, as opposed to the automatically generated data on physical activity through the PAT. Keeping a DFR can be time-consuming and burdensome, which could result in noncompliance or inadequate food logging and inadequate estimations of nutritional intake. Moreover, the complexity of the DFR used could impact compliance, since many persons reported on this topic in the online questionnaire. Finally, the tool used for assessing nutritional intake must be in line with the eating habits of the users to ensure compliance. In this case, the tool matched the eating habits of the Dutch study population but would be less suitable for a population with other eating habits.

### Strengths and Limitations

Strengths of this study include its prospective design and follow-up period with digital support in both the preoperative as well as the postoperative period for up to 10 weeks total. Moreover, we combined monitoring of physical activity and dietary protein intake. Both could be considered relevant for maintaining or building muscle mass and are thus related to clinical outcomes [11]. Since low-cost commercially available personal devices were used, they could be easily applied in similar circumstances by others.

Both an advantage and limitation to our study was the homogenous study population of persons with CRC up to 75 years of age. Selection bias has likely occurred as persons who have agreed to participate are more likely to find the self-monitoring tools acceptable and useful. Given the nature of our study’s population, bias could have occurred in selecting participants since our population is fairly young, technologically literate, and possibly more health conscious. This limits the generalizability of our findings. Finally, due to the small sample size, findings are preliminary and limited to usability and acceptability.

### Conclusions and Future Directions

The results of this study show that the use of a commercially available PAT is feasible, acceptable, and usable for the self-monitoring of physical activity in the perioperative setting. The use of a DFR to monitor protein intake was acceptable before surgery. A less extensive tool or a DFR with only a 4-day registration as an alternate [52] might increase compliance with protein intake monitoring. To our knowledge, this is the first study combining the monitoring of physical activity and dietary protein intake using low-cost commercially available tools in persons with surgical CRC. Future research should focus on integrating both monitoring tools and could include monitoring vital signs to give a complete picture of a person’s perioperative course. A large-scale data collection is necessary to validate the effects on clinical outcomes.

## Data Availability

The data sets generated and analyzed during the current study are available from the corresponding author on reasonable request.

## Abbreviations

CRC: colorectal cancer
CSAT: customer satisfaction score
DFR: digital food record
NPS: net promotor score
PAT: physical activity tracker
